# Dr. Bostock, on the Nitrat of Silver, in Epilepsy

**Published:** 1799-05

**Authors:** John Bostock

**Affiliations:** Liverpool


### *To the Editors of the Medical and Physical Journal.*

*Gentlemen,*

In the 2d Number of the Medical Journal, you direct the attention of your readers to the important ſubject of epilepſy. This dreadful diſorder, though not abſolutely incurable, ſo frequently reſiſts the beſt approved medicines, that every practitioner muſt think himſelf fully juſtified in attempting to introduce any new plan of cure, which may preſent a rational proſpect of ſucceſs. During my reſidence at Edinburgh, I had an opportunity of ſeeing the *nitrat of ſilver* uſed with very conſiderable advantage, which appeared the the more ſtriking, as contraſted with the total failure of the *cuprum ammoniacum,* a medicine whoſe credit reſts upon the very reſpectable authority of Cullen. In October laſt, my friend Dr. Cappe of York, who honoured me with a viſit in Liverpool, gave me an account of his ſucceſs in the uſe of the *argentum nitratum,* as detailed in p. 184, of your 2d Number. Theſe circumſtances determined me to give it a trial in the firſt fair inſtance which ſhould occur: only one caſe has hitherto preſented itſelf; but in this the *nitrat of ſilver* has proved of ſuch ſingular utility, that though but a *ſingle caſe,* I have been induced to offer it for admiſſion into your Journal. The following account is extracted partly from the Diſpenſary books, and partly from notes which I took at the time.

March 8. Owen Hughes, a boy aged 11, who works with a ſhoe-maker; of a pale complexion, and general unhealthy appearance, offered at our diſpenſary. About two months before he had been ſeized with the epilepſy; at firſt, one fit only occurred in the day; the diſorder however gradually increaſed in frequency and violence, till on the 8th of March, (the day that I firſt ſaw him) he had four, and on the 9th, five pretty ſevere fits. On the 9th he began the uſe of the *argentum nitratum*; gr. ij. of it were made into 40 pills, and he was ordered to take four of theſe pills, at two doſes (i. e. one fifth of a grain), in the courſe of the day.

March 15.—He had one fit on the 10th, but none ſince; he feels a ſlight ſenſation of heat in the ſtomach, after taking the pills; his appetite appears rather impaired. He was ordered to continue the pills as before, but to drink lb. ſs. of any fluid after each doſe: he was alſo directed to take ſcrup. i. of Peruvian bark twice in the day.

March 22.—He has had during the laſt week, three fits; his general health is as before: he now feels no uneaſineſs after taking the pills. He has had no warning of the approach of the three laſt fits; formerly he had a ſlight previous vertigo. I now ordered him to take three pills in the morning, and two in the afternoon, drinking after each doſe. The bark to be continued as before.

March 26.—He has had no more fits; the appetite is now improving. The medicines were ordered to be continued in the ſame manner.

April 12.—He has had no return of fits. His appetite and general health are now completely eſtabliſhed; he has worked at his trade for ten days, without inconvenience. The bowels have not been in the leaſt affected by the medicine; during the illneſs, the pulſe was little different from the healthy ſtate; and the other functions were in general little affected.

I ſhall

I ſhall indeed feel gratified, ſhould this caſe be in any degree the means of bringing into notice, a ſubſtance which may prove an efficacious remedy for a diſeaſe ſo dreadful in its appearance, and ſo injurious to the conſtitution, as epilepſy. Believe me, Gentlemen,

Your obedient ſervant,

*Liverpool, April* 15, 1799, JOHN BOSTOCK.

